# Clinical analysis of prophylactic cholecystectomy during gastrectomy for gastric cancer patients: a retrospective study of 1753 patients

**DOI:** 10.1186/s12893-019-0512-x

**Published:** 2019-05-14

**Authors:** Zhenhua Tan, Ping Xie, Hai Qian, Xing Yao

**Affiliations:** Department of Hepatobiliary Surgery, Huzhou Hospital, Zhejiang University School of Medicine (Huzhou Central Hospital), No. 198, Hongqi Road, Huzhou, 313000 Zhejiang Province China

**Keywords:** Cholecystolithiasis, Gastric cancer, Subtotal gastrectomy, Prophylactic cholecystectomy

## Abstract

**Background:**

Performance of gastrectomy in gastric cancer patients can lead to an increased incidence of cholecystolithiasis (CL) and a higher morbidity rate. However, the value of prophylactic cholecystectomy performed during gastric cancer surgery is still being debated.

**Methods:**

We carried out a retrospective study on patients with gastric cancer who underwent subtotal or total gastrectomy, with preservation of the gallbladder or simultaneous cholecystectomy from January 2010 to March 2018.

**Results:**

Cholecystolithiasis occurred in 152 of 1691 (8.98%) patients after gastric cancer surgery, with 45 (2.67%) patients undergoing subsequent cholecystectomy. Postoperative body mass index (BMI) decrease > 5% in 3 months was an independent risk factor for cholecystolithiasis [BMI decrease > 5%/≤5%: OR (95%CI): 1.812 (1.225–2.681), *p* = 0.003). Gastrectomy method and diabetes mellitus were independent risk factors for both cholecystolithiasis [gastrectomy method (no-Billroth I/Billroth I): OR (95%CI): 1.801 (1.097–2.959), *p* = 0.002; diabetes mellitus (yes/no): OR (95%CI): 1.544 (1.030–2.316), *p* = 0.036] and subsequent cholecystectomy [gastrectomy method (no-Billroth I/Billroth I): OR (95%CI): 5.432 (1.309–22.539), *p* = 0.020; diabetes mellitus (yes/no): OR (95%CI): 2.136 (1.106–4.125), *p* = 0.024]. Simultaneous cholecystectomy was performed in 62 of 1753 (3.5%) patients. The mortality and morbidity rates did not differ significantly between the combined surgery group and the gastrectomy only group (8.1% vs. 8.9 and 1.6% vs. 2.2%, respectively, *p* > 0.05).

**Conclusions:**

Prophylactic cholecystectomy may be necessary in gastric cancer patients without Billroth I gastrectomy and with diabetes mellitus. Simultaneous cholecystectomy during gastric cancer surgery does not increase the postoperative mortality and morbidity rates.

## Background

It is generally accepted that performance of gastrectomy in gastric cancer patients can lead to an increased incidence of cholecystolithiasis (CL) and a higher rate of morbidities that require secondary surgery [1], which may related with the complex interaction between sectioning of the nerve supply to the gallbladder and the change in cholecystokinin secretion [2, 3]. However, performance of routine prophylactic cholecystectomy during radical gastrectomy is still being debated [4]. The aim of this study was to investigate the correlative factors that contribute to gallstone formation and later cholecystectomy in patients with previous radical gastrectomy. The influences of simultaneous cholecystectomy on surgical mortality and morbidity of gastric cancer patients were also evaluated.

## Methods

### Patients

**Fig. 1:**
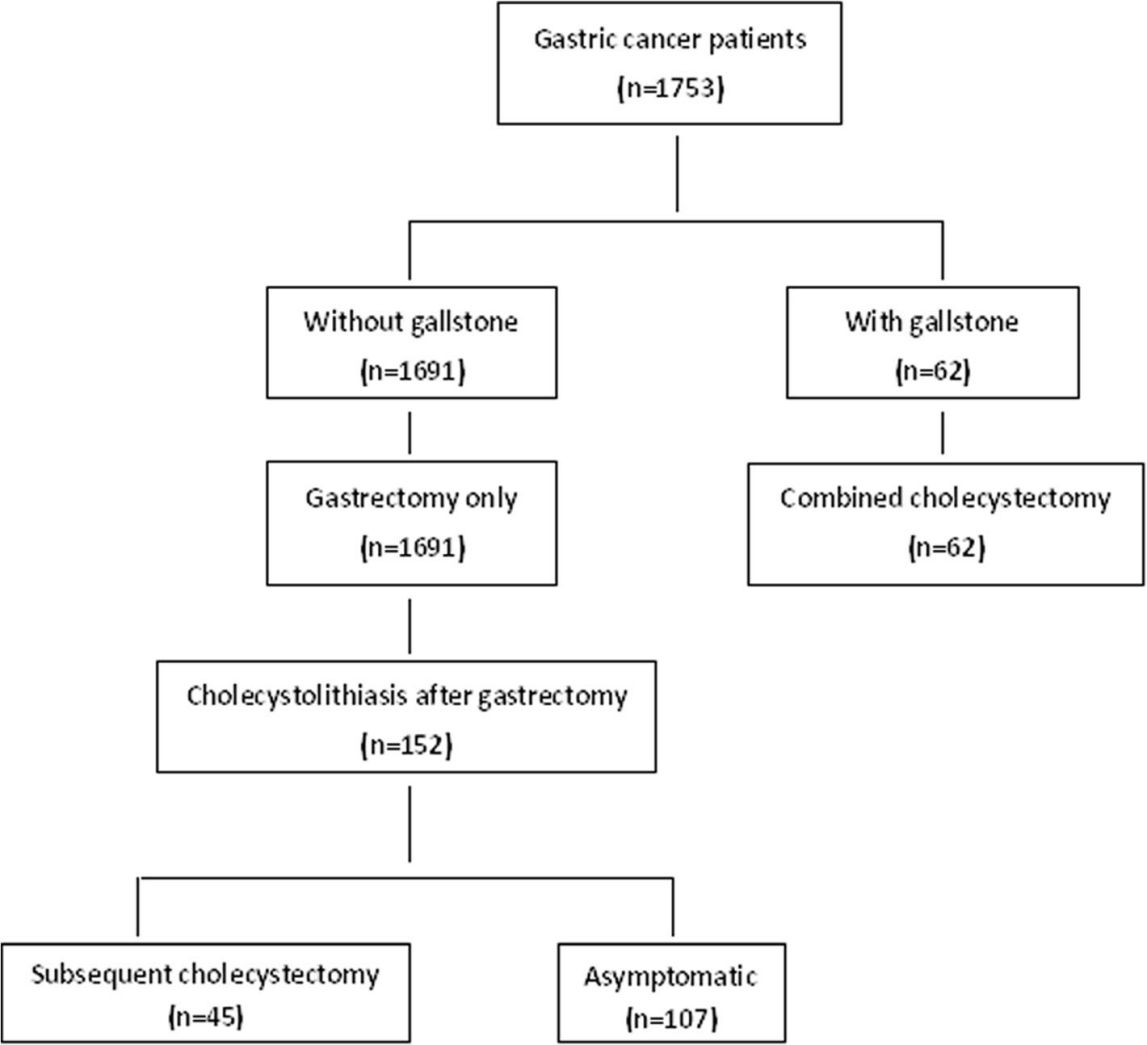
Flow chart showing the treatment of the gastric cancer patients with or without cholecystolithiasis

A total of 1753 gastric cancer patients underwent radical gastrectomy at the Department of Surgery, Huzhou Hospital, Zhejiang University School of Medicine (Huzhou Central Hospital), China from January 2010 to March 2018. The diagnosis of gastric cancer was confirmed by postoperative pathological examination of the resected specimen. The study was approved by the Ethics Committee of Huzhou Hospital, Zhejiang University School of Medicine, and written informed consent was obtained from all patients. In all patients, 1691 had no gallstones and preserved their gallbladders during radical gastrectomy. These patients were all followed-up and the median follow-up period after first surgery was 45 months (range: 11–96 months). Their gallbladders were routinely examined by ultrasonography or computed tomography during the postoperative period and 152 patients were diagnosed CL after gastric resection. Of these patients, 45 patients underwent a secondary cholecystectomy followed the following surgical indications: cholecystolithiasis combined with conservative treatment of uncontrollable cholecystitis (more than 3 times per month); complicated with acute pancreatitis; gallstones larger than 2 cm in diameter; cholecystolithiasis combined with gallbladder polyp; porcelain gallbladder; gallbladder atrophy; gallbladder filling stones; gallstones located in the gallbladder ampulla abdomen or gallbladder duct. Sixty-two patients with gallstones underwent simultaneous cholecystectomy during radical gastrectomy. A flow chart for the treatment of the gastric cancer patients with or without cholecystolithiasis is shown in Fig. 1.

### Study protocol

A total of 1691 gastric cancer patients without gallstones were evaluated retrospectively to determine the risk factors for cholecystolithiasis and later cholecystectomy. The clinicopathological characteristics of all patients were documented. The patients were grouped according to age (≤75/> 75 years), sex, rate of postoperative body mass index (BMI) decrease in 3 months [(preoperative BMI – postoperative BMI 3 months after surgery) / preoperative BMI × 100%] (≤5%/> 5%), cTNM stage, tumor differentiation grade, type of gastrectomy, extent of lymph node dissection, and presence of diabetes mellitus (yes/no). The tumor differentiation grades followed the criteria in the Edmondson–Steiner classification, and divided the patients into groups with well-differentiated tumors (Grade I), moderately-differentiated tumors (Grade II), and poorly-differentiated tumors (Grade III). Tumor stages were assigned according to the UICC TNM classification (7th edition). Then these groups were divided again to postoperative cholecystolithiasis (yes/no) and subsequent cholecystectomy (yes/no) groups for further study. The clinical outcomes of the 1691 patients who underwent gastrectomy only and the 62 patients who underwent combined surgery were reviewed retrospectively and documented, including postoperative mortality, postoperative wound infection, anastomotic leak, duodenal stump leak, gastric stasis, intraabdominal abscess, hemorrhage, pulmonary infection, diarrhea, postoperative pancreatitis, and cholangitis. The mortality and morbidity rates in the two groups were compared to determine the influence of simultaneous cholecystectomy on surgical safety of radical gastrectomy.

### Statistical analysis

All data were prepared and compiled using SPSS software (version 19.0 for Windows; IBM Corp., Armonk, NY, USA). The Chi-square test and Fisher’s exact test were used for quantitative data. Risk factors were analyzed by logistic regression analysis. A *p*-value <0.05 was considered statistically significant.

## Results

### Patient characteristics

The characteristics of the 1691 patients who did not undergo simultaneous cholecystectomy during gastric resection are shown in Table 1. The patients comprised 1222 males and 469 females, with a male-to-female ratio of 2.61:1 and an age range of 23–84 years (median age: 61 years). Regarding the gastric cancer stages, stage I was diagnosed in 534 (31.6%) patients, stage II in 712 (42.1%) patients, and stage III in 445 (26.3%) patients. For tumor differentiation grades, Grade I was present in 617 (36.5%) patients, Grade II in 523 (30.9%) patients, and Grade III in 551 (32.6%) patients. Billroth I reconstruction was performed in 334 (19.8%) patients, Billroth II in 876 (51.8%) patients, and total gastrectomy and Roux-en-Y reconstruction in 481 (28.4%) patients. D1 lymph node dissections were carried out in 264 (15.6%) patients, and D2 lymph node dissections in 1427 (84.4%) patients. A total of 152 patients had gallstones after gastrectomy and the incidence of cholecystolithiasis was 8.98% (152/1691). Forty-five patients underwent later cholecystectomy, giving a subsequent operation rate of 2.67% (45/1691).

**Table 1:** Clinicopathological characteristics of 1691 gastric cancer patients who underwent gastrectomy without cholecystectomy (median age: 61.0 ± 10.5 years; age range: 23–84 years)

Variable	No. of patients (%)
Gender	
Male	1222 (72.3%)
Female	469 (27.7%)
TNM stage	
Stage I	534 (31.6%)
Stage II	712 (42.1%)
Stage III	445 (26.3%)
Tumor differentiation grade	
Grade I	617 (36.5%)
Grade II	523 (30.9%)
Grade III	551 (32.6%)
Method of gastrectomy	
Billroth I	334 (19.8%)
Billroth II	876 (51.8%)
Roux-en-Y	481 (28.4%)
Lymph node dissection	
D1	264 (15.6%)
D2	1427 (84.4%)
Cholecystolithiasis after gastrectomy	152 (8.98%)
Subsequent cholecystectomy after gastrectomy	45 (2.67%)

### Risk factors fo2r cholecystolithiasis after gastrectomy

The median interval from gastrectomy to development of cholecystolithiasis was 37.5 months (range: 8.0–94.7 months) and 152 patients had gallstones after gastrectomy, giving an incidence of cholecystolithiasis of 8.98%. Univariate analyses showed that postoperative BMI decrease >5% in 3 months, method of gastrectomy (Roux-en-Y, Billroth II), and diabetes mellitus were significantly associated with occurrence of gallstone formation (*p* = 0.002, *p* = 0.018, and *p* = 0.038, respectively). Logistic regression analysis identified these clinicopathological features as risk factors for occurrence of cholecystolithiasis after gastrectomy [BMI decrease (> 5%/≤5%): OR (95%CI): 1.812 (1.225–2.681), *p* = 0.003; gastrectomy method (no-Billroth I/Billroth I): OR (95%CI): 1.801 (1.097–2.959), *p* = 0.002; diabetes mellitus (yes/no): OR (95%CI): 1.544 (1.030–2.316), *p* = 0.036], as shown in Table 2.

**Table 2:** Correlations between the clinicopathological characteristics of 1691 gastric cancer patients and postoperative cholecystolithiasis

Variables	Postoperative cholecystolithiasis		χ2	*p* value	Odds ratio (95% CI)	*p* value
	Yes (*n* = 152)	No (*n* = 1539)				
Age (years)						
>75	34	412	1.381	0.288		
≤75	118	1127				
Gender						
Male	105	1117	0.846	0.393		
Female	47	422				
BMI decreased rate						
≤5%	35	541	9.057	0.002*	1	0.003*
>5%	117	998			1.812 (1.225–2.681)	
TNM stage						
Stage I+ II	114	1132	0.149	0.699		
Stage III	38	407				
Tumor differentiation grade						
Grade I + II	96	1044	1.378	0.240		
Grade III	56	495				
Method of gastrectomy						
Billroth I	19	315	5.541	0.018*	1	0.002*
No-Billroth I	133	1224			1.801 (1.097–2.959)	
Lymph node dissection						
D1	30	234	2.157	0.159		
D2	122	1305				
Diabetes mellitus						
No	118	1297	4.471	0.038*	1	0.036*
Yes	34	242			1.544 (1.030–2.316)	

*p < 0.05

### Risk factors for subsequent cholecystectomy after gastrectomy

A total of 45 (2.67%) patients required a subsequent cholecystectomy after gastrectomy. Of these, 34 patients underwent open cholecystectomy and 11 patients underwent laparoscopic cholecystectomy. Univariate analyses showed that method of gastrectomy (Roux-en-Y, Billroth II) and diabetes mellitus were significantly associated with occurrence of later cholecystectomy (*p* = 0.007 and *p* = 0.038, respectively). Logistic regression analysis identified these two clinicopathological features as risk factors for occurrence of subsequent cholecystectomy [gastrectomy method (no-Billroth I/Billroth I]: OR (95%CI): 5.432 (1.309–22.539), *p* = 0.020; diabetes mellitus (yes/no): OR (95%CI): 2.136 (1.106–4.125), *p* = 0.024], as shown in Table 3.

**Table 3:** Correlations between the clinicopathological characteristics of 1691 gastric cancer patients and subsequent cholecystectomy

Variables	Subsequent cholecystectomy		χ2	*p* value	Odds ratio (95% CI)	*p* value
	Yes (*n* = 45)	No (*n* = 1646)				
Age (years)						
>75	11	435	0.089	0.865		
≤75	34	1211				
Gender						
Male	32	1190	0.031	0.867		
Female	13	456				
BMI decreased rate						
≤5%	9	567	4.071	0.055		
>5%	36	1079				
TNM stage						
Stage I+ II	32	1214	0.158	0.732		
Stage III	13	432				
Tumor differentiation grade						
Grade I + II	32	1108	0.287	0.633		
Grade III	13	538				
Method of gastrectomy						
Billroth I	2	332	6.834	0.007*	1	0.020*
No-Billroth I	43	1314			5.432 (1.309–22.539)	
Lymph node dissection						
D1	10	254	1.533	0.213		
D2	35	1392				
Diabetes mellitus						
No	32	1383	5.346	0.038*	1	0.024*
Yes	13	263			2.136 (1.106–4.125)	

*p < 0.05

### Postoperative morbidity and mortality rates in the simultaneous cholecystectomy group and gastrectomy only group

Of the total 1793 gastric cancer patients, 62 (3.5%) patients with gallstones underwent simultaneous cholecystectomy during gastric resection. The mortality and morbidity rates did not differ significantly between the combined surgery group and the gastrectomy only group (8.1% vs. 8.9 and 1.6% vs. 2.2%, respectively, *p* > 0.05), as shown in Table 4.

**Table 4:** Postoperative morbidity and mortality rates in the simultaneous cholecystectomy group and the gastrectomy only group

	Simultaneous cholecystectomy (*n* = 62)	Gastrectomy only (*n* = 1691)	χ^2^	*p* value
Postoperative morbidity	8.1% (4.9)	8.9% (145)	0.021	0.997
Wound infection	8.1% (5)	8.7% (147)	0.030	0.991
Hemorrhage	3.2% (2)	3.3% (55)	0.021	0.998
Anastomotic leak	6.4% (4)	7.2% (121)	0.045	0.997
Duodenal stump leak	1.6% (1)	1.7% (29)	0.031	0.992
Gastric stasis	12.8% (8)	14.8% (251)	0.179	0.855
Intraabdominal abscess	3.2% (2)	3.1% (52)	0.022	0.998
Pulmonary infection	6.4% (4)	6.7% (114)	0.008	0.998
Diarrhea	17.7% (11)	17.4% (295)	0.004	1.000
Postoperative cholangitis	8.1% (5)	9.6% (162)	0.159	0.828
Postoperative pancreatitis	11.3% (7)	13.2% (224)	0.200	0.848
Postoperative mortality	1.6% (1)	2.2% (38)	0.111	1.000

## Discussion

Patients undergoing gastrectomy, especially radical gastrectomy, have a higher incidence of postoperative gallstone formation [5, 6]. The underlying mechanisms of this phenomenon are unclear. The complex interaction between the supply of cholecystic nerves and the secretion of cholecystokinin may play an important role. In gastric cancer surgery, to achieve better lymph node dissection, the liver branch of the vagus nerve is inevitably damaged. Deletion or damage to the vagus nerve branch can result in impaired gallbladder emptying, which in turn may lead to gallstone formation [7, 8]. Also, radical gastrectomy usually involves Billroth II and Roux-en-Y techniques, which eliminate food passing through the duodenum, and may reduce secretion of cholecystokinin and gallbladder activity, promoting cholestasis and gallstone formation [2]. Although several studies confirmed that the incidence of gallstones after gastrectomy is significantly higher than that in patients without upper gastrointestinal surgery, whether there is a need for preventive gallbladder resection in gastric cancer surgery remains controversial. The incidence of gallstones and symptomatic cholelithiasis requiring cholecystectomy after gastric cancer surgery is still relatively low [9]. In the present study, we observed cholecystolithiasis after gastrectomy in only 8.98% of patients, which was lower than the rates of 13–22% reported in previous studies [2, 4, 6]. We also found a low incidence of subsequent cholecystectomy of only 2.67% of patients. Based on these data, we believe that not all patients undergoing gastrectomy require routine preventive cholecystectomy. However, our identification of risk factors leading to gallstone formation and subsequent cholecystectomy is important and can help surgeons develop a rational surgical treatment strategy to avoid subsequent surgery or over-treatment.

Various clinical or surgical factors appear to be responsible for the development of cholecystolithiasis or the requirement for subsequent cholecystectomy after radical gastrectomy. Regarding surgical methods, several studies reported that the incidence of gallstone formation was affected by the gastrectomy procedure, especially the type of gastrointestinal reconstruction [10]. A recently study found that in patients who underwent distal gastrectomy, there are no significant difference in gallstones incidence between performance of Billroth I, Billroth II, or Roux-en-Y anastomosis, but most research results indicated that the incidence of gallstones is higher in patients with Biroth II and Roux-en-Y anastomosis (duodenal exclusion) [11] and the patients who underwent total gastrectomy with resection of the esophagus and complete vagus nerve stem [12].

Lymph node dissection during radical gastrectomy is another possible risk factor for gallstone formation and subsequent cholecystectomy. Some studies have shown that the degree of lymph node dissection has an influence on gallstone formation [8]. More involved lymph node dissections are associated with a higher incidence of gallstones and acute cholecystitis following subsequent surgery than with limited or standard lymph node dissection. D2 lymph node dissection includes dissection along the hepatoduodenal ligament, which may lead to gallstones or cholecystitis by affecting gallbladder emptying, but routine concomitant cholecystectomy and D2 lymph node dissection remains controversial [13]. Surgical techniques, age, BMI reduction after gastrectomy, and diabetes are candidate risk factors for gallstone formation after gastrectomy. Rapid weight loss can also lead to mobilization of cholesterol stored secondary to gallbladder bile deposition. Supersaturation, as well as cholestasis secondary to bile duct inflammation, can promote gallstone formation [14].

The effect of diabetes on gallstones is multifactorial; diabetes leads to neuritis and neuromyopathy. In diabetes, increased oxidative stress at low heme oxygenase-1 levels and decreased insulin and insulin-like growth factor-1 signaling lead to loss of interstitial cells of Cajal, which results in abnormal gallbladder emptying and promotes gallstone formation [15]. Diabetes mellitus is also a well-known risk factor for cholelithiasis, which can lead to subsequent cholecystectomy [16].

In this retrospective study, we applied Fisher’s exact test and logistic regression analysis to identify the risk factors for gallstone formation and subsequent cholecystectomy after radical gastrectomy, and found that postoperative BMI decrease > 5% in 3 months, gastrectomy method (Roux-en-Y or Billroth II), and diabetes mellitus were independent risk factors for cholecystolithiasis. Roux-en-Y or Billroth II reconstruction and diabetes mellitus were also independent risk factors for subsequent cholecystectomy. These results indicated that prophylactic cholecystectomy may be necessary in patients with Roux-en-Y or Billroth II gastrectomy and with diabetes mellitus. H However, some studies reported that the postoperative complication incidences of simultaneous cholecystectomy during gastrectomy is higher than which of late cholecystectomy [12]. In our study, there was no significant increase in postoperative morbidity or mortality in patients who underwent cholecystectomy during radical gastrectomy.

In summary, prophylactic and simultaneous cholecystectomy during gastric resection for gastric cancer patients is safe, but does not need to be a routine operation for all patients undergoing radical gastrectomy; however, this approach may be recommended in patients undergoing Roux-en-Y or Billroth II reconstruction or in patients with diabetes mellitus.

The limitations and possible biases of this study are lack of randomization as a retrospective study, which may cause selective bias. In some groups, the small number of patients may make detection of small differences between study groups unreliable.

## Conclusions

This study showed that prophylactic cholecystectomy may be necessary in gastric cancer patients without Billroth I gastrectomy and with diabetes mellitus. Simultaneous cholecystectomy during gastric cancer surgery does not increase the operative mortality and morbidity rates.

## Abbreviations

BMI: body mass index
CL: cholecystolithiasis
